# New substitutions on NS1 protein from influenza A (H1N1) virus: Bioinformatics analyses of Indian strains isolated from 2009 to 2020

**DOI:** 10.1002/hsr2.626

**Published:** 2022-04-25

**Authors:** Syeda Lubna, Suma Chinta, Prakruthi Burra, Kiranmayi Vedantham, Sibnath Ray, Debashree Bandyopadhyay

**Affiliations:** ^1^ Birla Institute of Technology and Science, Pilani, Hyderabad Campus Hyderabad Telangana India; ^2^ Nagarjuna Innovation Center Hyderabad India

**Keywords:** cellular binding partner, H1N1 virus, host–pathogen interactions, NS1 protein, viral pathogenicity

## Abstract

**Background and Aims:**

Nonstructural (NS1) protein is mainly involved in virulence and replication of several viruses, including influenza virus A (H1N1); surveillance of the latter started in India in 2009. The objective of this study was to identify the new substitutions in NS1 protein from the influenza virus A (H1N1) pandemic 2009 (pdm09) strain isolated in India.

**Methods:**

The sequences of NS1 proteins from influenza A(H1N1) pdm09 strains isolated in India were obtained from publicly available databases. Multiple sequence alignment and phylogeny analyses were performed to confirm the “consistent substitutions” on NS1 protein from H1N1 (pdm09) Indian strains. Here, “consistent substitutions” were defined as the substitutions observed in all the sequences isolated in a year. Comparative analyses were performed among NS1 Indian sequences from A(H1N1) pdm09, A (H1N1) seasonal and A(H3N2) strains, and from A (H1N1) pdm09 global strains.

**Results:**

Eight substitutions were identified in the NS1 Indian sequence from the A(H1N1) pdm09 strain, two in RBD, five in ED, and one in the linker region. Three new substitutions were reported in this study at NS1 sequence positions 2, 80, and 155, which evolved within 2015–2019 and became “consistent.” These new substitutions were associated with conservative paired substitutions in the alternative domains of the NS1 protein. Three paired substitutions were (i) D2E and E125D, (ii) T80A and A155T, and (iii) E55K and K131E.

**Conclusions:**

This study indicates the continuous evolution of NS1 protein from the influenza A virus. The new substitutions at positions 2 and 80 occurred in the RNA binding and eIF4GI binding domains. The D2E substitution evolved simultaneously with the E125D substitution that involved viral replication. The third new substitution at position 155 occurred in the PI3K binding domain. The possible consequences of these substitutions on host–pathogen interactions are subject to further experimental and computational verification.

## 1 INTRODUCTION

Amidst the current worldwide threat by coronavirus disease 2019 (COVID‐19), also known as severe acute respiratory syndrome coronavirus 2 (SARS‐COV2 virus) pandemic, the influenza virus remains a threat, according to the report by the World Health Organization (WHO) (WHO|Influenza update—385). Both influenza and SARS‐COV2 viruses cause respiratory diseases. The influenza A(H1N1) virus‐infected patients are often susceptible to COVID‐19.^1^, ^2^, ^3^, ^4^, ^5^ Hence, proper characterization of host–pathogen (influenza) interactions is crucial for better understanding of both the diseases. Influenza virus A has caused four major pandemics across the world—(i) Spanish flu caused by H1N1 (1918–1919), (ii) Asian flu by H2N2 (1957–1958), (iii) Hong Kong flu by H3N2 (1968–1969), and (iv) swine flu by A(H1N1) pandemic 2009 (pdm09) strain (2009–2010).^6^ The A(H1N1) pdm09 strain has shown increased virulence due to its swine transmutability to human host.^7^, ^8^ The segmented genome of the influenza A virus helps the genetic reassortment of the virus with the possible emergence of new subtypes.^9^ New subtypes often evade the host immune system more efficiently, leading to increased viral spread and pandemic outbreaks.

Virologic surveillance is crucial in controlling the influenza virus in various nations. Surveillance tracks the genetic nature of the virus, thus understanding the relatedness of the flu vaccine and the nature of the circulating virus (Weekly US Influenza Surveillance Report|CDC). Currently, the flu strain circulating in the United States is A(H3N2). Similarly, the influenza virus is being tracked in India by the National Center for Disease Control (https://ncdc.gov.in/index4.php?lang=1%26level=0%26linkid=119%26lid=276). The pandemic outbreak of the influenza virus escalated in 2015 in India, in contrast to the worldwide report by WHO. The pandemic outbreak in 2015 in India was more severe than in 2009.^10^ Considering the recent epidemic of the influenza virus in India, it is essential to understand the possible genetic drift and shift of the virus to predict future pandemics possibilities.

The protein of choice in this study was the NS1 nonstructural protein, as it invades the host immune system and blocks different host signaling pathways. NS1 protein modulates host antiviral responses and promotes viral replication. Hence, it plays a pivotal role in viral pathogenesis.^11^ The NS1 is a multifunctional protein^12^ involved in posttranslational regulation of the influenza virus life cycle. It binds virion RNA,^12^, ^13^, ^14^, ^15^ poly(A)‐containing RNA,^15^ human U6 snRNA,^16^ and many more viral and cellular proteins.

NS1 protein has two major domains, the N‐terminal RNA binding domain (RBD) (residues 1–73) and the C‐terminal effector domain (ED) (residues 84 to the end).^17^, ^18^, ^19^ The RBD protects the virus against the antiviral state induced by interferon alpha/beta by blocking the activation of the 2′−5′ oligo (A) synthetases/RNase L pathway.^11^, ^12^, ^20^ Several host factors (RNA/protein) interact with NS1 RBD and ED domains. Some of these host factors are protein kinase R (PKR),^11^, ^12^, ^21^ retinoic‐acid inducible gene I,^12^, ^21^, ^22^ oligoadenylate synthetase,^11^, ^12^, ^20^ protein activator EIF2AK2, P^ACT21^ tripartite motif‐containing 25,^11^, ^12^, ^20^, ^21^, ^22^, ^23^ and NF90.^21^ The question asked in this study was whether new mutations in NS1 protein from Indian isolates have emerged recently. If such mutations have evolved, what are the possible consequences of those mutations on host–pathogen interactions? The first question was addressed using multiple sequence alignment (MSA), and phylogenetic analyses of NS1 proteins from H1N1 (pdm09) strains circulating in India. The results were compared with those obtained from H1N1 seasonal strains, H3N2 strains, and H1N1 (pdm09) strains circulating worldwide. The answer to the second question was intuitive, based on the sequence positions of NS1 mutations belonging to various cellular‐binding partners. The hypothesis generated from the second question is subject to further experimental and computational verifications.

## 2 METHODOLOGY

### 2.1 Generation of data sets for NS1 protein sequences obtained from different influenza virus strains in India and worldwide

The data sets were generated from influenza A virus NS1 sequence data, available on the public database NCBI (Influenza virus database—NCBI; https://www.ncbi.nlm.nih.gov)^24^ and restricted database, GISAID EpiFlu database (GISAID Initiative).^25^, ^26^

#### 2.1.1 NS1 Indian data set for H1N1 seasonal and pdm09 strains

NCBI Influenza Virus Resource was searched with the following selection criteria, (i) H1N1 strains isolated from India, (ii) protein name NS1, (iii) human host, (iv) laboratory strains excluded, and (v) sequences deposited on or before December 30, 2020. The above search criteria resulted in 218 sequences (pdm09 and seasonal strains). Selection criteria from the EpiFlu database were following: (i) influenza virus type A, (ii) only H1N1 strains, (iii) from the human host, (iv) location—India, Asia, (vi) lineage—pdm09, and (vii) collection dates till December 30, 2020. The above search criteria resulted in 165 NS1 sequences from the EpiFlu database. Additionally, we searched for the “seasonal” lineage in the EpiFlu database, with three alterations in the search criteria, (i) lineage—“seasonal,” (ii) no specific collection dates, and (iii) complete NS1 protein (in additional filters for the search criteria, “only complete”), resulting into 0 NS1 sequences. Three hundred and eighty‐three NS1 sequences were retrieved from the influenza A(H1N1) virus isolated in India from 2007 to 2020.

The redundancy removal tool CD‐HIT (CD‐HIT Suite [weizhongli‐lab. org])^27^, ^28^ removes redundant sequences based on a given sequence identity cutoff following the heuristic approach. The sequence identity cutoff used was 100%. The final Indian data set consists of 95 NS1 sequences (79 H1N1 pdm09 and 16 seasonal H1N1 strains).

#### 2.1.2 NS1 global data set for H1N1 seasonal and pdm09 strains

Similar search criteria, as described above, were used to construct the global data set from the NCBI database, with the only difference—location “India” was being removed. Sixteen thousand and one sequences were obtained from NCBI. Similarly, 25,594 and 918 sequences were obtained for H1N1 pdm09 and seasonal H1N1 strains, respectively, from the EpiFlu database. The total number of NS1 sequences obtained worldwide was 42,513.

#### 2.1.3 NS1 Indian data set for A(H3N2) strain

A(H3N2) Indian strains were obtained from NCBI and EpiFlu databases, using similar search criteria as A(H1N1); the only difference was in the strain name, “H3N2,” instead of “H1N1.” Ninety‐nine sequences were obtained, 26 from NCBI and 73 from the EpiFlu database. After redundancy removal, there were 22 sequences in the final NS1 data set from A(H3N2) strain.

### 2.2 Construction of phylogenetic trees and evolution of amino acid substitutions

MEGAX software^29^, ^30^ was used to construct the phylogenetic trees for influenza A virus NS1 proteins from Indian isolates. As a prerequisite to phylogenetic tree construction, multiple sequence alignment was performed using CLUSTALW.^31^ Following pairwise alignment parameters were used, (i) gap opening penalty value—10.00 and (ii) gap extension penalty value—0.10. For multiple alignment parameters, the gap opening penalty value was set to 10.00, and the gap extension penalty value to 0.20. No negative matrix weight was used. Delay divergence cutoff was 30%. Phylogenetic trees were constructed for (i) the final Indian A(H1N1) data set (*n* = 95), (ii) the final Indian A(H1N1) pdm09 subset (*n* = 79), and (iii) the final Indian A(H3N2) data set (*n* = 22). The evolutionary history was inferred using the Maximum Likelihood (ML) method based on the Jones–Taylor–Thornton (JTT) matrix‐based model.^32^ ML is a statistical method to infer probability distribution and assign probabilities to predicted phylogenetic trees. JTT substitution model was used to assess the likelihood of particular substitutions. No additional options were used to handle gaps and missing data (i.e., all sites were used). Initial heuristic search tree(s) were obtained automatically by applying Neighbor‐Join and BioNJ algorithms to a matrix of pairwise distances estimated using a JTT model.^33^ Five hundred steps of bootstrap replication were performed to construct each phylogenetic tree. The final topology was selected based on the superior log‐likelihood value. Visualization of the phylogenetic tree was done using MEGAX.

### 2.3 Host factor (protein/RNA)–pathogen (H1N1 NS1 protein) interaction study

NS1 interacting partners from human host proteins and their sites of interactions were identified from Google Scholar and PubMed database using the search criteria “Influenza A virus,” “NS1 protein,” and “protein interaction.” Five such interacting partners were identified for NS1 protein, namely, (i) PI3K,^12^, ^21^, ^34^, ^35^, ^36^, ^37^, ^38^, ^39^ (ii) poly(A)‐binding protein II (PABII),^12^, ^17^, ^40^, ^41^, ^42^, ^43^ (iii) double‐stranded RNA (dsRNA),^17^, ^18^, ^22^, ^43^, ^44^, ^45^, ^46^, ^47^ (iv) nuclear localization signal 1 (NLS1),^11^, ^12^, ^13^, ^19^, ^45^, ^46^, ^47^ and (v) eukaryotic translation initiation factor 4 G 1 (eIF4GI).^12^, ^17^, ^21^, ^42^

Solved crystal structures for NS1 protein from H1N1 strain were available only for two complexes in global isolates, (i) NS1 RBD domain complexed with dsRNA (PDB ID: 2ZKO) and (ii) NS1 ED complexed with PI3K host protein (PDB ID: 3L4Q). All the interaction sites were retrieved from the secondary database PDBSUM.^48^

## 3 RESULTS

### 3.1 Divergence of the NS1 protein from H1N1 virus across India, over the period 2009–2020, based on phylogenetic trees

**Figure 1:**
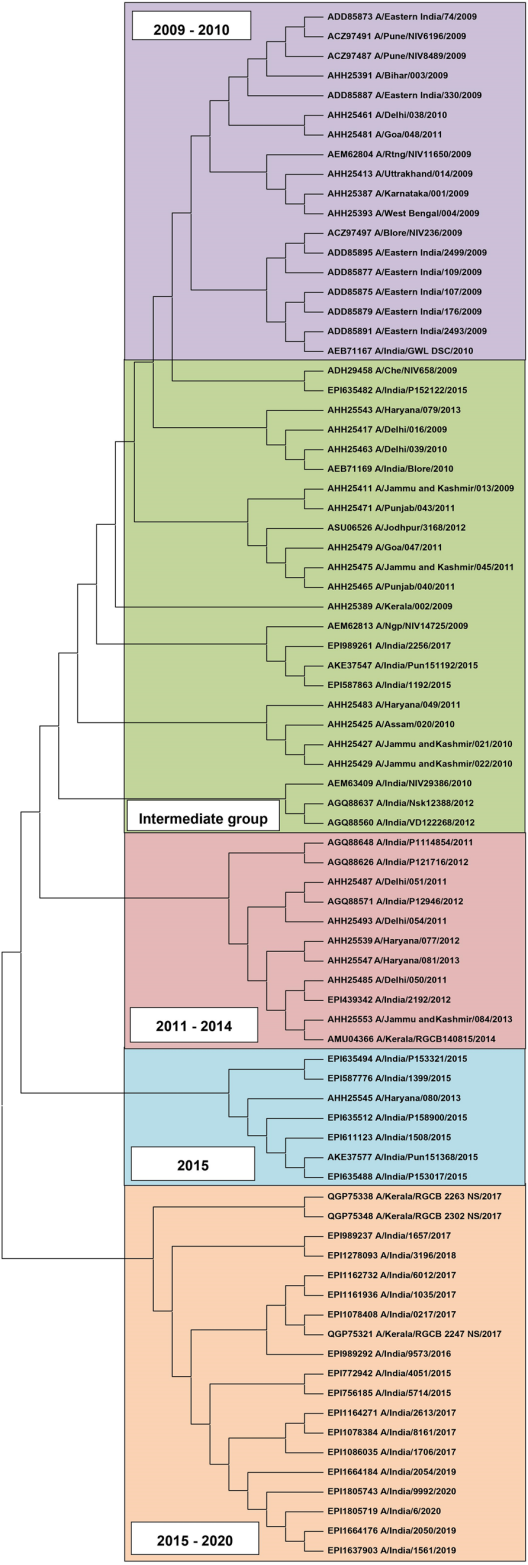
Phylogenetic tree for A(H1N1) nonstructural (NS1) protein sequences from the final Indian pandemic 2009 (pdm09) sub‐data set (*n* = 79). The final trees are drawn to scale, with branch lengths measured in terms of the number of substitutions per site, after 500 steps of bootstrapping. Evolutionary analyses were conducted using MEGA X software. The tree has the highest log‐likelihood value of −1420.39.

A phylogenetic tree was constructed for NS1 sequences from Indian A(H1N1) final data set (*n* = 95). The seasonal strains appeared separate from pdm09 strains (Figure S1). A second phylogenetic tree was constructed for NS1 sequences from Indian A(H1N1) pdm09 subset (*n* = 79) (Figure 1). The sequences in this tree showed chronological evolution based on the proximity of the branches. Five groups were observed in this tree, (i) 2009–2010, (ii) intermediate, (iii) 2011–2014, (iv) 2015, and (v) 2015–2020. The “intermediate” group contained strains from 2009 to 2017. Although the sequences from the intermediate group belong to pdm09 strains, they carry signatures similar to seasonal strains (Table 1). Moreover, the “intermediate” group perpetually partitioned the pdm09 strains before and after 2010.

**Table 1:** Common amino acids at different residue positions of NS1 protein among A(H1N1) seasonal strain and intermediate subgroup of A(H1N1) pdm09 strain.

Residue position	Seasonal strain (16)	Intermediate subgroup (24)	pdm09 strain (55)
4	H (13)/N (3)	H (1)/N (23)	N (55)
53	N (14)/D (2)	N (1)/Y(1)/D (22)	D (55)
108	K (16)	K (3)/R (21)	R (55)
123	I (16)	I (6)/V (18)	V (55)
129	I (16)	I (4)/L(2)/A(3)/V (15)	V (55)
143	N (12)/T (4)	N (1)/T (23)	T (55)
205	S (16)	S(4)/N (20)	N (30)/S (25)

*Note*: The number of NS1 sequences in each strain and the intermediate subgroup are shown in parentheses. Amino acids are represented by one‐letter code.

Abbreviations: NS1, nonstructural protein 1; pdm09, pandemic 2009.

### 3.2 Recent substitutions deduced from multiple sequence analyses, phylogeny, and evolutionary rate

We have identified 33 sequence positions that varied between seasonal and pandemic strains (Table S1). These changes (e.g., XIY) were denoted as substitution (Y) of the consensus amino acid (X) at sequence position I. For example, in the “intermediate” group, the consensus amino acid, glutamic acid (E), was substituted by glutamine (Q) at sequence position 55 (E55Q). One more frequently used term, “consistent substitution,” is defined here as substitution observed in all the sequences isolated in a year. For example, the N205S substitution was *consistent* from 2013 onwards (Table 2). This substitution was also reported earlier in the strains from India.^49^ The L90I substitution was first observed in 2011 (in 10 out of 23 sequences) and became *consistent* in 2014; the substitution was also reported earlier from India.^49^

**Table 2:** Chronological evolution of substitutions in NS1 protein sequence positions, obtained from A(H1N1) pdm09 strains.

Sequence position	2	55	80	90	125	131	155	205
Consensus residues	D	E	T	L	E	K	A	N
Substitution	E	Q/G/K	A	I	‐	Q/E	T	S
2009 (82)	‐	Q (3), G (4)	‐	‐	‐	‐	‐	‐
2010 (32)	‐	Q (20)	‐	‐	‐	‐	‐	‐
2011 (23)	‐	Q (6)	‐	I (10)	‐	‐	‐	S (17)
2012 (41)	‐	‐	‐	I (33)	‐	Q (2)	‐	S (14)
2013 (21)	‐	Q (1), K (3)	‐	I (19)	‐	E (6)	‐	**S (21)**
2014 (5)	‐	K (4)	‐	**I (5)**	‐	E (4)	‐	**S (5)**
2015 (69)	E (3)	**K (69)**	‐	**I (69)**	G (5), D (3)	**E (69)**	‐	**S (69)**
2016 (10)	E (8)	**K (10)**	‐	**I (10)**	D (8)	**E (10)**	‐	**S (10)**
2017 (36)	E (15)	**K (36)**	‐	**I (36)**	**D (36)**	**E (36)**	‐	**S (36)**
2018 (6)	**E (6)**	**K (6)**	‐	**I (6)**	**D (6)**	**E (6)**	‐	**S (6)**
2019 (10)	**E (10)**	**K (10)**	**A (10)**	**I (10)**	**D (10)**	**E (10)**	**T (10)**	**S (10)**
2020 (12)	**E (12)**	**K (12)**	**A (12)**	**I (12)**	**D (12)**	**E (12)**	**T (12)**	**S (12)**

*Note*: Consensus residues are shown in the second row and the substitutions, in chronological order, are shown in the following rows, if any. In case, there is no change from the consensus residues, it is designated by “‐.” Amino acids are represented by one‐letter code. Number of sequences is shown in parentheses. Consistent substitutions (substitutions observed in all the sequences isolated in a year) are shown in bold.

Abbreviations: NS1, nonstructural protein 1; pdm09, pandemic 2009.

**Figure 2:**
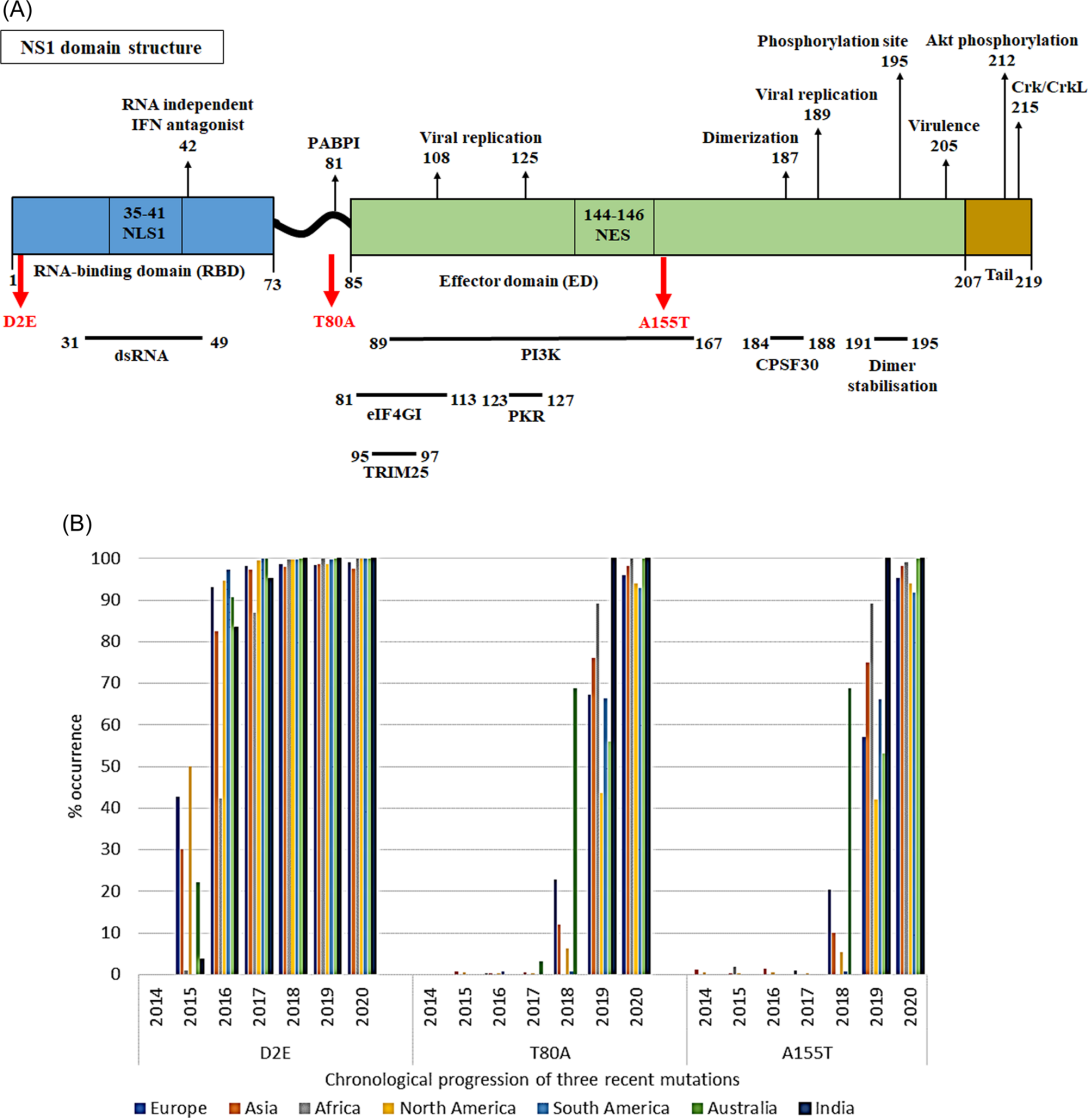
(A) Domain organization of nonstructural (NS1) protein from A(H1N1) influenza virus and its interacting partners (shown by black horizontal lines), most recent substitutions (shown by red vertical arrows) and functional residues (shown by black vertical arrows) in different domains. (B) Chronological progression of three recent substitutions in global sequences, along with those in Indian sequences. Percent (%) occurrence = (the number of sequences showing a particular substitution/total number of sequences) × 100.

Three conservative paired substitutions, namely (i) E55K and K131E, (ii) D2E and E125D, and (iii) T80A and A155T, were observed in Indian sequences. The presence of conservative substitutions (paired substitutions) has not been reported earlier, although conservative substitutions were frequently observed in densely packed proteins where two interacting amino acids exchange their positions.^50^ Here, paired substitutions are defined by a pair of amino acids exchanging their positions among RBD and EDs of NS1 protein, thus leaving the total number of amino acid types unchanged. The first paired substitution (E55K and K131E) was observed in 2013 and was *consistent* in 2015. The single substitution at 55th position (G/Q/K) was first noted in 2009. The other substitution, K131E, was observed in 2012, and finally, the substitution became *consistent* in 2015 (Table 2). The second paired substitution, D2E, and E125D, was first observed in 2015 and became *consistent* in 2017. The third paired substitution, T80A and A155T, were observed in 2019 and became *consistent* in 2019 only. The significance of these three paired substitutions is yet to be explored. Five out of the eight substitutions (E55K, L90I, E125D, K131E, and N205S) observed in the current study from Indian isolates were reported earlier in global strains.^51^ This study had three unique substitutions, namely, D2E, T80A, and A155T (Table 2). The D2E substitution on NS1 protein from A(H1N1) pdm09 was reported earlier, only once from Russia.^52^ To further confirm our observation, we had analyzed the global sequence, isolated from 2009 to 2020. The analysis showed that these three unique substitutions in NS1 protein (Figure 2A) identified from India were simultaneously detected in global NS1 sequences (Figure 2B).

Next, we asked whether these three new substitutions were unique to A(H1N1) strains. Hence, we have analyzed the NS1 sequences available from the A(H3N2) strain circulating in India. The average pairwise sequence similarity computed across the strains of influenza viruses circulating in India was 87.5% (Table 3). The similarities among the strains were higher than those across the strains. Despite having high sequence similarity across the strains, the residue positions 2, 80, and 155 varied in A(H1N1) strain from 2009 to 2020, in contrast to A(H3N2) strain where these three sequence positions (Asp2, Thr80, and Ala155) showed no changes (Figure S2). However, the phylogenetic tree based on the final A(H3N2) NS1 Indian data set showed significant variation (Figure S3). The set of sequence positions substituted in the A(H3N2) NS1 Indian data set was different from those in the A(H1N1) NS1 Indian data set(Table S2). These results suggested that the three new substitutions in NS1 protein were unique to A(H1N1) pdm09 strains.

**Table 3:** Average pairwise NS1 sequence similarity (%) computed among different strains of influenza virus from India using CLUSTALW.

Influenza A virus strains	*N*	Similarity (%)
A(H1N1), A(H3N2)	117	87.5
A(H1N1) (pdm09 & seasonal)	95	91.6
A(H1N1) pdm09	79	97.0
A(H1N1) seasonal	16	95.0
A(H3N2)	22	97.1

*Note*: Number of sequences (*N*) in each final data set is shown in the table.

Abbreviations: NS1, nonstructural protein 1; pdm09, pandemic 2009.

To ascertain the above substitutions in NS1 protein from Indian A(H1N1) pdm09 strains, we have estimated position‐specific evolution rate. These rates were scaled so that the average evolutionary rate across all the sites was one. Thus, the sites showing a rate less than one evolved slower than the average evolutionary rate and vice versa.

**Figure 3:**
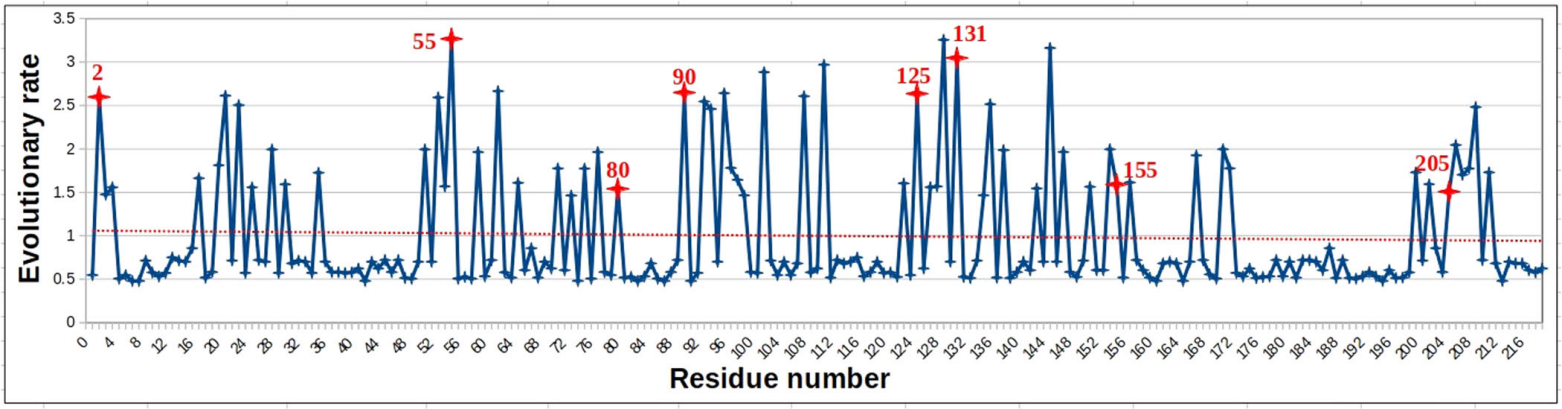
Evolutionary rate analysis for nonstructural protein 1 (NS1) sequences, Indian A(H1N1) pdm09 subset (*n* = 79), using MEGAX software. The evolutionary rate was highlighted for eight *consistent substitutions* identified in this study. The average evolutionary rate is depicted by the trend line. pdm09, pandemic 2009.

Fifty‐seven positions were identified with an evolutionary rate greater than one (Figure 3). All these positions showed a higher evolutionary rate due to the difference in the type of substituted amino acids, as per the substitution matrix in the JTT model. However, all the eight *consistent* substitutions identified (Table 2) have an evolutionary rate greater than two, except two of the new substitutions, T80A and A155T, which emerged only in 2019 but their evolutionary rate is still greater than one (Figure 3). The third new substitution, D2E (evolved in 2015), showed an evolutionary rate greater than two.

### 3.3 Host factor (protein/RNA) interactions with influenza A(H1N1) NS1 protein involving the new substitutions identified in this study

**Figure 4:**
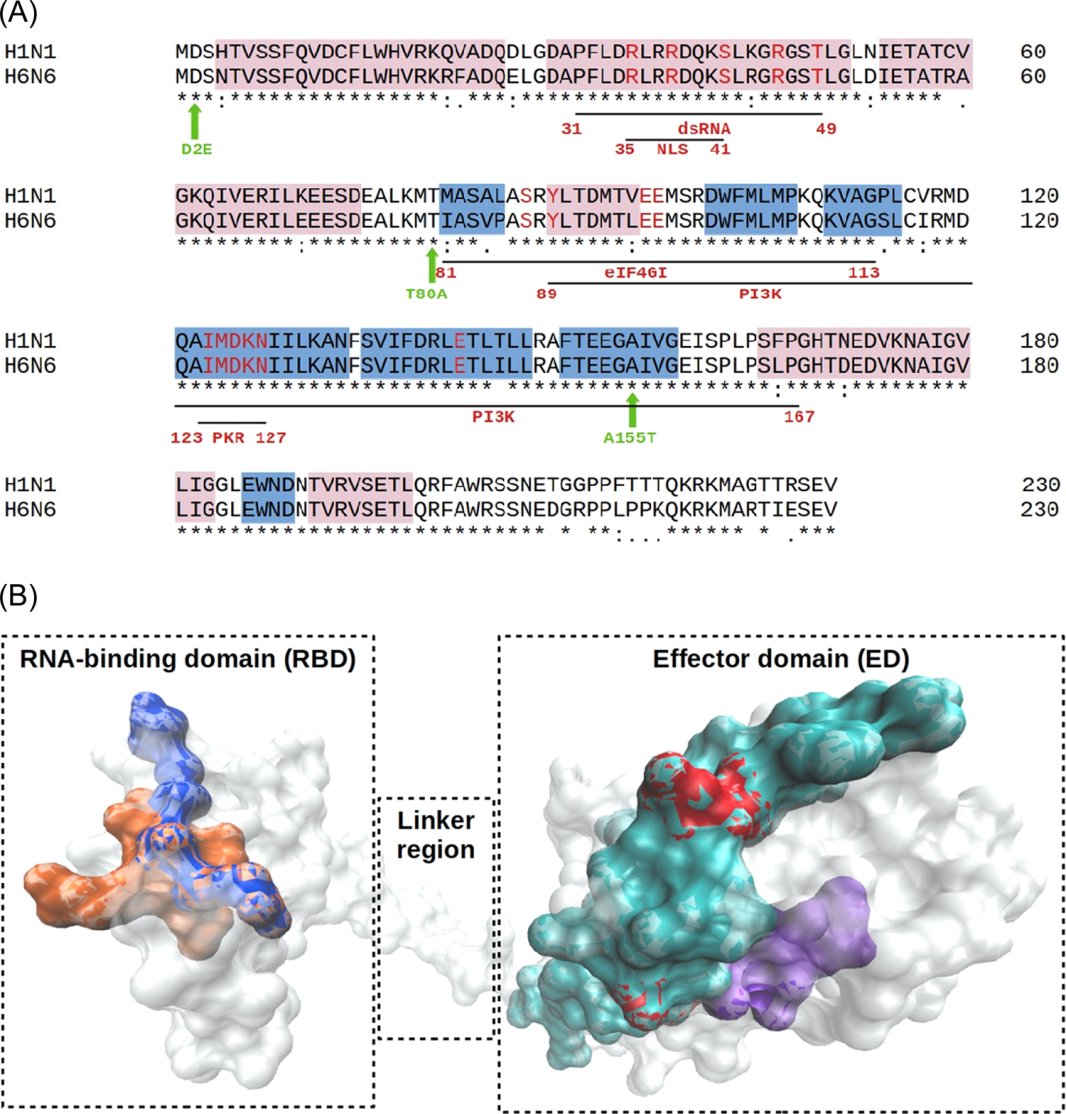
(A) Nonstructural (NS1) protein sequence alignment between A(H1N1) and A(H6N6) influenza virus strains. Residues involved in host interactions are shown by black horizontal lines indicating start and end residue positions. Host interaction partners are named in red. The three new substitutions identified on A(H1N1) pandemic 2009 (pdm09) strain in this study are shown by green vertical arrows. Secondary structures, alpha helix, and beta strands are depicted by pink and blue colors, respectively. (B) Key residues of influenza virus depicted on the crystal structure obtained from influenza virus H6N6 strain (PDB ID: 6NRL). The host dsRNA binding residues are depicted in blue, nuclear localization signal (NLS) binding depicted in orange, EIF4G binding residues depicted in cyan, protein kinase R (PKR) binding residues depicted in violet, and PI3K binding residues are depicted in red.

As crystal structures are not available for complete NS1 protein from the H1N1 strain, the interacting residues are depicted on the crystal structure obtained from the NS1 protein of the H6N6 strain. The NS1 protein sequences from these two strains are mostly conserved (Figure 4A). The dsRNA binding residues are buried within the protein, whereas those from nuclear localization signal binding are mostly exposed on the protein surface (Figure 4B). Similarly, residues 123–127 from the ED domain of influenza virus NS1 proteins (those that interact with the PKR domain) are buried within the protein core. The residue positions, 81–113, identified on the eIF4GI subdomain interact with the host elongation factor (eIF4GI). The residue position, 81, was also involved in the interaction between the PABPI domain of NS1 protein with the host protein.^42^

## 4 DISCUSSION

### 4.1 Domains and critical residues of NS1 proteins involved in different viral replications and blocking of host replications

Multifunctional NS1 proteins are involved in the replication of most of the viral subtypes; for example, NS1 protein from parvovirus minute virus of mice assists the viral genome to locate at the cellular sites of DNA damage.^53^ The NSP1 protein of SARS‐COV2 interacts with messenger RNA (mRNA) export receptor heterodimer NXF1‐NXT1 and prevents binding of NXF1 to mRNA export adaptor and docking of NXF1 at the nuclear pore complex.^54^ The NS1 homodimer is responsible for zika virus replication that can be potentially prevented by disrupting the dimer formation via disulfide reduction.^55^, ^56^ These observations indicate the direct involvement of NS1 proteins in the prevention of host replication and promoting viral replication. However, the structural organization of NS1 proteins vary across the viral subtypes—(a) NSP1 from SARS‐COV2 contains a β‐barrel domain,^54^ (b) NS1 from zika virus contains a dominant homodimeric β ladder segment,^55^, ^56^ (c) NS1 from dengue virus, similar to zika virus, contains three defined domains, namely wing domain, β‐barrel domain, and β roll domain,^57^, ^58^ and (d) NS1 from influenza (H1N1) has two defined domains—RBD and ED (Figure 2A). The critical residues of influenza virus NS1 protein—Arg35, Arg38, Ser42, Arg46, and Thr49—are involved in host dsRNA binding (PDB ID: 2ZKO). Additional residues, Thr5, Asp29, Asp34, Asp39, and Lys41, interact with host dsRNA via water‐mediated interactions (fig. 1 and 2 of Cheng et al.^59^). Residues, 35–41, on NS1 protein from influenza virus binds to nuclear localization signal (NLS1) subdomain that participates in nuclear import of NS1 protein via classical alpha/beta nuclear import pathway.^19^, ^45^, ^46^, ^47^ The key residues and the interaction patterns with the partner molecules vary in other viral subtypes. In the case of the zika virus NS1 protein, membrane, and antibody binding regions are exposed on the protein surface. The membrane‐binding residues are primarily hydrophobic, and antibody binding residues are mostly polar.^56^

In this study, we identified three new substitutions (2015 onwards) on NS1 protein from influenza virus A(H1N1) pdm09 circulating in India, at positions 2, 80, and 155. Residue positions 2 and 80 are located in the RNA‐binding and near‐eIF4GI binding domains, respectively. The NS1, a multifunctional protein, binds to 5′‐UTR of viral mRNA and eIF4GI–host translation initiation factor; thus, involved in the enhancement of viral mRNA translation. The PABP1 protein binds to NS1 sequence positions 1–81. Collectively, NS1, eIF4GI, PABP1, and viral mRNA recruit 43S ribosomal complexes to viral mRNA.^42^ Hence, substitutions at these two positions (2 and 80), presumably, would alter the viral mRNA translation. One notable observation was residue position 2 has a conserved paired substitution with residue position 125 from 2016 onwards (Table 2). Residue position 125 (along with residue positions 108 and 189) is also involved in viral replication.^48^, ^49^, ^60^ Thus, both residues 2 and 125, part of the conserved paired substitutions, are involved in viral replication. Residue position 155 is located in the PI3K binding domain of NS1 protein. The NS1 protein binds to the multimeric PI3K protein in its inactive form (regulatory [p85‐β] subunit attached to the catalytic [p110] subunit) and releases p85‐β subunit leading to subsequent activation of PI3K protein. The activated PI3K promotes PIP3 production, which activates the Akt pathway.^35^, ^38^, ^61^ Hence, the substitution in the PI3K binding domain is thought to affect a cascade of cellular pathways.

## 5 CONCLUSION

Influenza virus is frequently mutated, leading to many genetic drifts and shifts since its first outbreak in 1918, thus leading to yearly vaccination against flu as per CDC (Influenza (Flu)|CDC). Hence, virologic surveillance is essential to control influenza viral infection. Due to the recent outbreak of the influenza A virus in India, monitoring is required for amino acid substitutions on viral proteins. This study aimed to identify the recent substitutions (2015 onwards) on NS1 proteins from Indian A(H1N1) pdm09 strains. The methods applied were multiple sequence alignment, phylogeny, and substitution analyses on different influenza virus strains, Indian and global. Eight substitutions on NS1 protein from the A(H1N1) pdm09 strain were identified. Three new substitutions (2015 onwards)—D2E, T80A, and A155T (unique to H1N1 strain only)—were for the first time reported here. These three new substitutions were in NS1 domains for cellular interactions with various host factors: residue position 2 involves RNA binding and position 80 involves eIF4G1 binding and recruitment of viral mRNA to 43S ribosomal complexes. We, therefore, hypothesized that these substitutions on NS1 protein alter cellular binding. However, this hypothesis is subject to validation by experimental and computational studies, such as molecular dynamic studies on host–pathogen protein complexes with different mutant models.

## AUTHOR CONTRIBUTIONS

**Suma Chinta**: Data curation; formal analysis; methodology; validation; visualization; writing—original draft. **Prakruthi Burra**: Data curation; formal analysis; methodology; validation; visualization; writing—original draft. **Kiranmayi Vedantham**: Data curation; formal analysis; methodology; validation; visualization. **Sibnath Ray**: Data curation; formal analysis; methodology. **Debashree Bandyopadhyay**: Conceptualization, formal analysis, investigation, methodology, project administration, supervision, validation, writing—review & editing.

## CONFLICTS OF INTEREST

The authors declare no conflicts of interest.

## TRANSPARENCY STATEMENT

This manuscript is an honest, accurate, and transparent account of the study being reported; that no important aspects of the study have been omitted.

## WEB RESOURCES

World Health Organization data of influenza cases and COVID‐19 cases where retrieved from: https://www.who.int/influenza/surveillance_monitoring/updates/latest_update_GIP_surveillance/en/ Influenza virus database of NCBI was used to get the influenza NS1 protein sequences, retrieved from: https://www.ncbi.nlm.nih.gov/genomes/FLU/Database/nph-select.cgi GISAID EpiFlu database was used to retrieve influenza NS1 protein sequences from: https://platform.gisaid.org/epi3/cfrontend#4feb29 CD‐HIT Suite: Biological Sequence Clustering and Comparison was used as redundancy tool: http://weizhongli-lab.org/cdhit_suite/cgi-bin/index.cgi.

## Supporting information

Supporting information.

## Data Availability

The data analyzed here is publicly available at Influenza virus database—NCBI (https://www.ncbi.nlm.nih.gov) and GISAID Initiative.
