# Supplementary material for: New substitutions on NS1 protein from influenza A (H1N1) virus: Bioinformatics analyses of Indian strains isolated from 2009 to 2020
Source: Health Sci Rep. 2022 Apr 25;5(3):e626. doi: 10.1002/hsr2.626 (PMC9059196; doi:10.1002/hsr2.626)

Table S1: Variation in amino acids in 33 different sequence positions of H1N1 NS1 protein among seasonal and pandemic strains

| Sl.no. | Consensus sequence | Sequence position | Pandemic | Seasonal |
| --- | --- | --- | --- | --- |
| 1 | M | 6 | M | V |
| 2 | I | 18 | I | V |
| 3 | R | 21 | R | Q |
| 4 | F | 22 | F | V |
| 5 | N | 25 | N | Q |
| 6 | G | 26 | G | E/D |
| 7 | N | 48 | N | S |
| 8 | L | 59 | L | C/R |
| 9 | W | 67 | W | R |
| 10 | S | 74 | S | D |
| 11 | T | 76 | T | A |
| 12 | R | 78 | R | K |
| 13 | I | 81 | I | M |
| 14 | V | 84 | V | A |
| 15 | P | 85 | P | L |
| 16 | T | 86 | T | A |
| 17 | S | 91 | S | T |
| 18 | L | 95 | L | V |
| 19 | I | 112 | I | A |
| 20 | L | 119 | L | M |
| 21 | N | 139 | N | D |
| 22 | L | 166 | L | F |
| 23 | V | 178 | V | I |
| 24 | G | 189 | G | D |
| 25 | N | 197 | N | T |
| 26 | I | 198 | I | L |
| 27 | D | 207 | D | N |
| 28 | R | 211 | R | G |
| 29 | S | 213 | S | P |
| 30 | L | 214 | L | F |
| 31 | P | 215 | P | T |
| 32 | P | 216 | P | T |
| 33 | E | 217 | E | T/K |

Table S2: Chronological evolution of substitutions in NS1 protein sequence positions, obtained from H3N2 strains. Consensus sequence and the substitutions, in chronological order, are shown. Amino acids are shown in one letter code. Number of sequences are shown in parenthesis. Consistent substitutions (substitutions observed in all the sequences isolated in a year) are shown in bold.

| Consensus sequence -> | E | A | E | N | K |
| --- | --- | --- | --- | --- | --- |
| Residue position -> | 26 | 56 | 71 | 207 | 229 |
| substitutions -> | K | S/E/T | G | H | E |
| 2007 (15) | K (1) | S (1) | G (1) |  | E (1) |
| 2008 (18) |  |  |  |  |  |
| 2009 (17) |  |  |  |  |  |
| 2011 (2) |  | E (2) |  |  |  |
| 2016 (6) | **K (6)** | T (1) |  |  | **E (6)** |
| 2017 (3) | **K (3)** | **S (3)** | **G (3)** |  | **E (3)** |
| 2018 (30) | **K (30)** | **S (30)** | **G (30)** |  | **E (30)** |
| 2019 (3) | **K (3)** | **S (3)** | **G (3)** |  | **E (3)** |
| 2020 (5) | **K (5)** | **S (5)** | **G (5)** | **H (5)** | **E (5)** |

Figure S1: Phylogenetic tree of the final H1N1 NS1 Indian dataset (n=95).


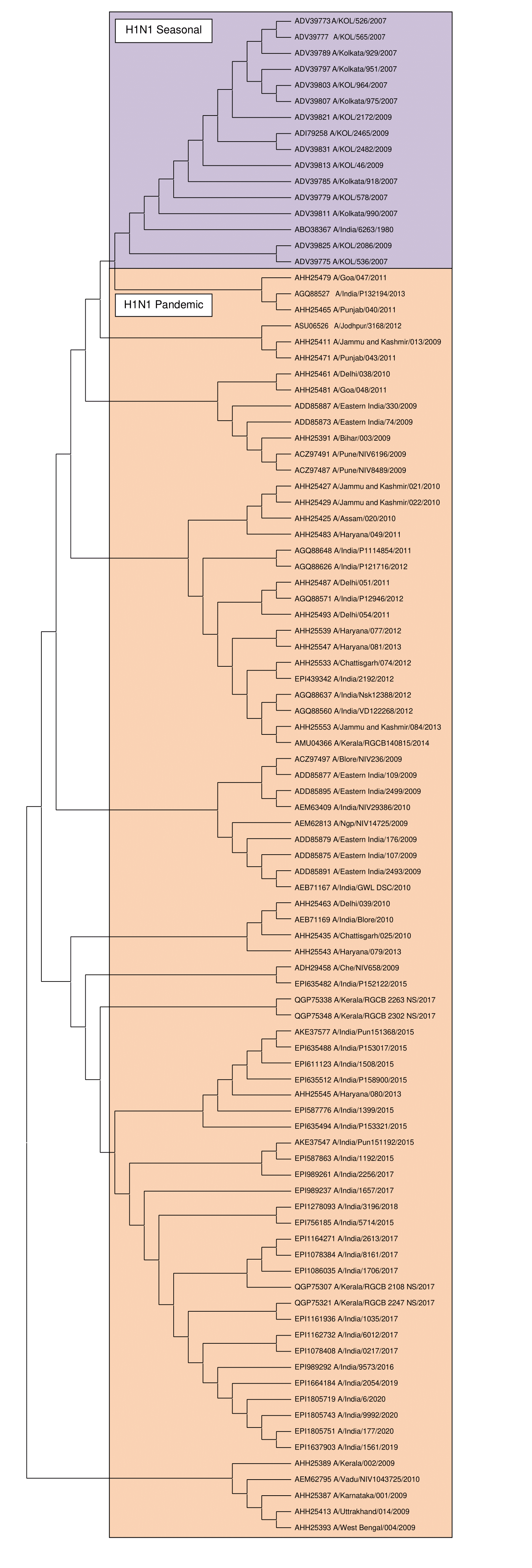


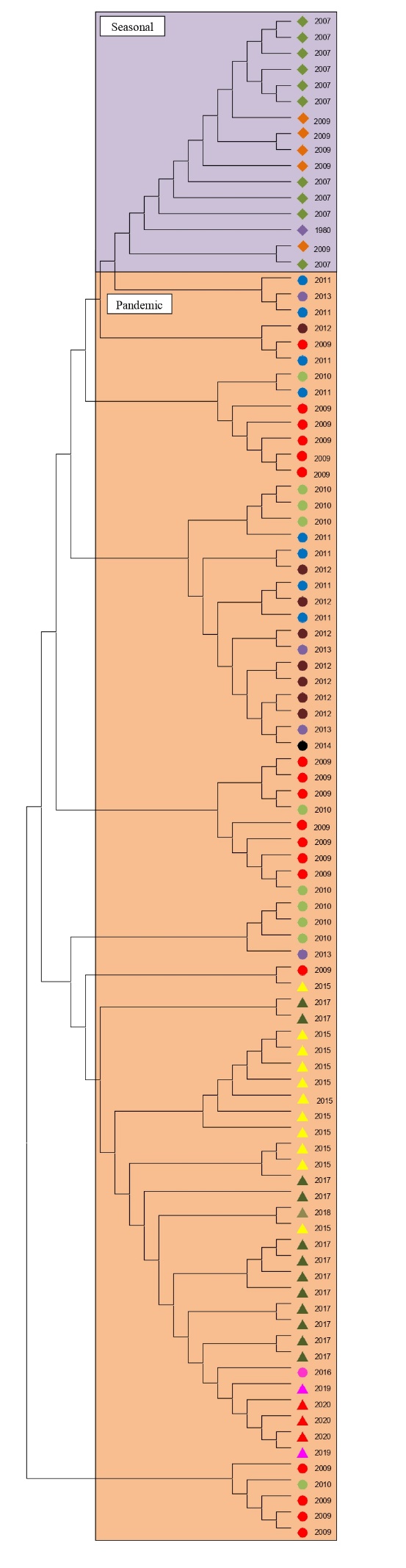


Figure S2: Multiple sequence alignment for A(H3N2) NS1 final dataset (n=22). The cyan color shows the conserved residue positions and the white color shows variable residue positions. Three residue positions, 2, 80 and 155 (new substitutions in A(H1N1) strain) are depicted in black. Consensus sequences are given below the alignment.


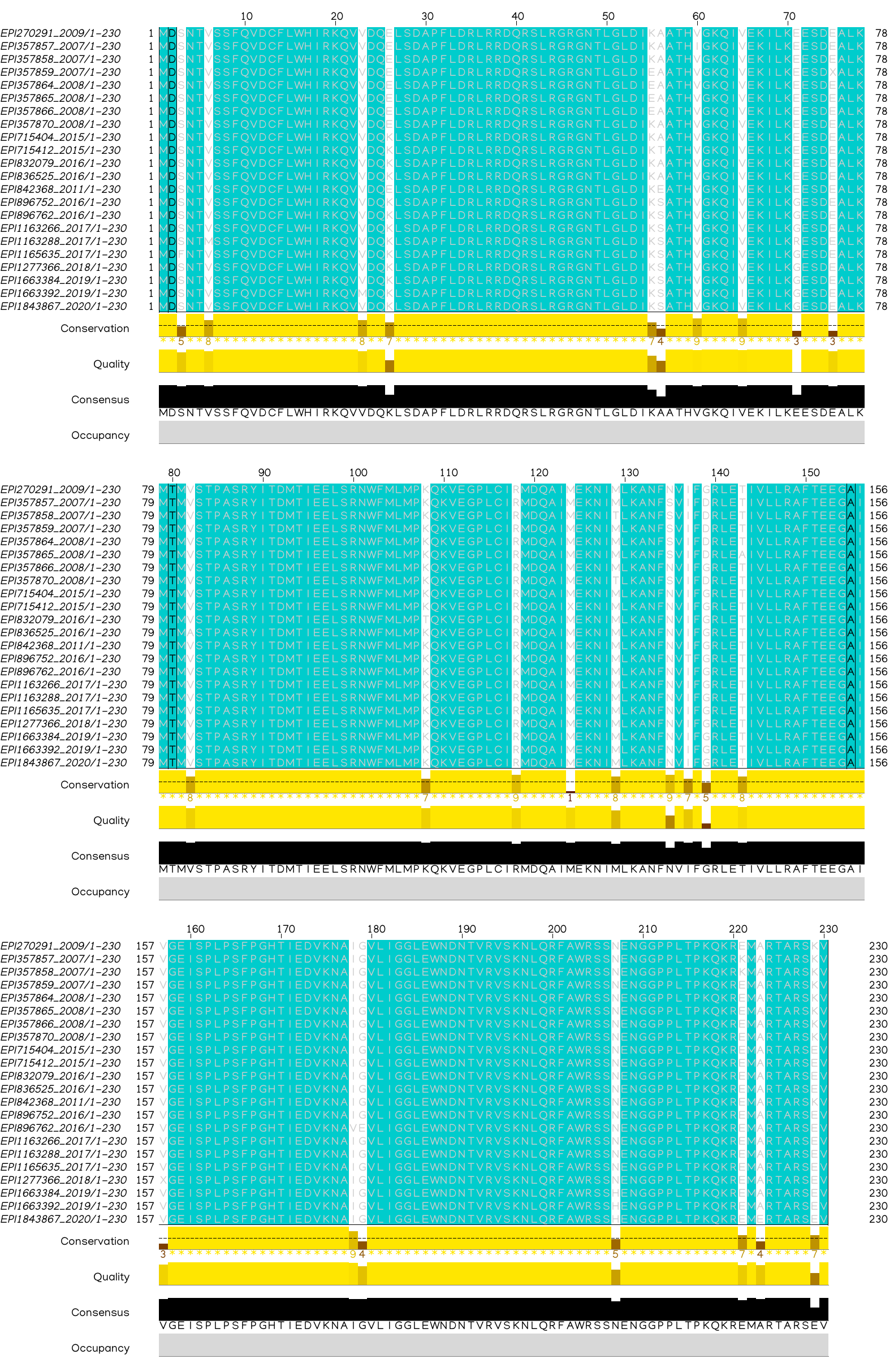


Figure S3: Phylogenetic tree of the final H3N2 NS1 Indian dataset (n=22)


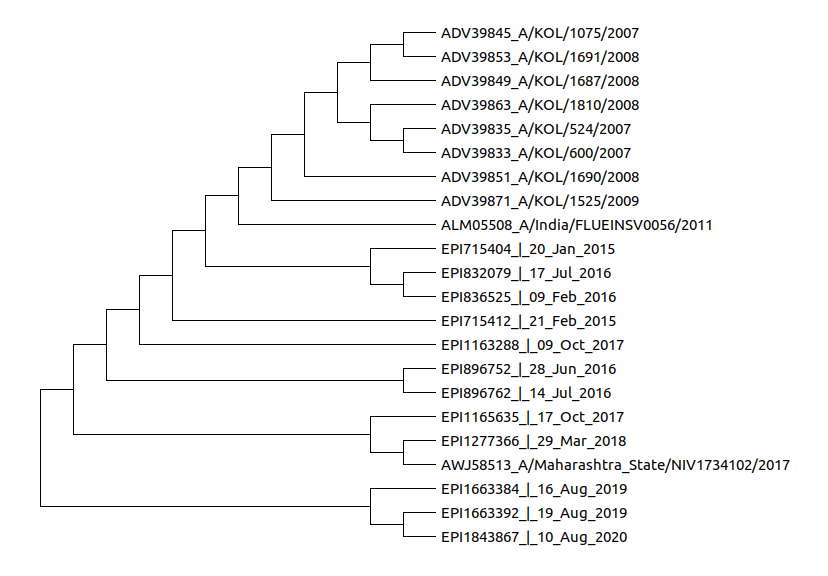

Supplement: Supplementary file 1 — Supporting information. [file HSR2-5-e626-s001.doc]
